# Withdrawal: Identification and classification of surface defects for digital twin models of the workpiece

**DOI:** 10.1371/journal.pone.0310791

**Published:** 2024-09-16

**Authors:** The PLOS ONE Editors

This article [1] was published in error by PLOS after the authors requested withdrawal. Therefore, *PLOS ONE* has withdrawn this article [1] and removed the article contents from the journal website. The publisher apologizes for the error.
